# Physiological responses of race car drivers in authentic and simulated motor-racing

**DOI:** 10.3389/fspor.2025.1498686

**Published:** 2025-03-06

**Authors:** Justin Holland, Megan Davis, David Ferguson

**Affiliations:** ^1^ School of Exercise and Nutrition Science, Queensland University of Technology, Brisbane, QLD, Australia; ^2^ Department of Kinesiology, Michigan State University, East Lansing, MI, United States

**Keywords:** automobile racing, driver science, thermoregulation, simulated driving, environmental physiology

## Abstract

**Objectives:**

The aim of the present investigation was to determine the influence of G-loading and psycho-emotional stress and competitive pressures on driver physiology between authentic and simulated racing during similar environmental conditions (e.g., a hot cockpit).

**Methods:**

Authentic racing data was collected during the 2018 “Sahlen's 6 h at the Glen” race, where five male drivers (age = 38.0 ± 5.1 y, driving years = 8 y) competed in the IMSA GTD class in 1 h stints over the course of the race. In the simulated race, the same drivers wore a full protective outfit to replicate the attire worn in the authentic race for 60 min in an environmental controlled room that matched authentic racing. During authentic and simulated racing physiological measures of heart rate (HR), breathing rate, physiological strain index (PSI), skin temperature and core temperature were recorded.

**Results:**

In the final 50 min higher (*P* < 0.05) physiological demands were observed in core temperature, PSI, and breathing rate for authentic racing compared to simulated racing. HR in the final 50 min was higher (*P* < 0.001) in authentic racing (159 ± 23 beats·min^−1^) to simulated racing (112 ± 19 beats·min^−1^) with no increase in heart rate in the first 10 min of simulated racing. In authentic racing skin temperature was higher (*P* < 0.001) in the first 10 min compared to simulated racing however, in the final 50 min there was no difference (*P* = 0.928) observed.

**Conclusions:**

G-loading and psycho-emotional stress lead to considerable increases in metabolic work and physical stress in authentic racing compared to simulated racing. A racing simulator does not generate the physical loads to drive the car or the psycho-emotional stress and competitive pressure of an authentic racing event.

## Introduction

Automobile racing is a globally popular sport with a similar viewing audience to football (American Soccer) (1). Success in automobile racing is dependent on driver skill/fitness and engineering of the vehicle for optimal speed and handling (2). To achieve harmony between driver and vehicle for increased performance, racing teams travel to racetracks to engage in non-competitive testing sessions. However, testing is becoming cost prohibitive and in some racing leagues teams are restricted on the number of tests per year (3), as such teams are increasingly relying on racing simulators to optimize performance. Racing simulators have been used for over 30 years to improve car performance, help drivers learn the racetrack, and in recent years simulator competition has become a surrogate for authentic racing (especially during the time of COVID-19) (4). Popular media implies simulators are directly correlated to actual racing, however there is no empirical data to support the media's claims (5). As driver physical and mental fitness can influence on track performance (6), if simulators do not elicit similar physiological responses to authentic racing the resulting disconnect between authentic racing and simulated racing could hinder performance development.

Automobile racing is a physically demanding sport. During competition drivers' heart rates will be 65%–85% of heart rate maximum due to psycho-emotional stress and competitive pressure, increased subcutaneous blood flow, and oxygen consumption of contracting skeletal muscle in thermally challenging environments (6–11). Heightened psycho-emotional states are a key contributor to elevations in heart responses particularly in the response to high g-force loads and the competitive racing environment (11, 12). Elevated heart rate responses during a racing situation compared to solo racing (12) and during qualifying compared to free practice (13) having been observed in the literature alluding to the psycho-emotional demands of competition. Similarly, observations have been made in military pilots during critical time events of flight e.g., takeoff and landing attributable to higher cognitive loads (14).

Certain aspects of authentic racing are not fully replicated in the simulator, including the intense psychological demands, the physical forces from impacts and track irregularities, and the gravitational loads experienced by the driver. Additionally, the perceptual experience in a simulator differs from real-world racing, as it lacks some of the sensory and physiological cues that influence performance on the track. Racing cars utilize aerodynamics to increase the grip the race car has in the corners so that the car can travel faster. A consequence of these aerodynamic techniques is the increased gravitational forces placed on the drivers, which can range to 2 to 5 times the force of gravity (3). A gap in the literature is the relationship between physiological responses of authentic race cars and racing simulators. Understanding such a relationship will allow teams to optimize car development to account for physiological responses of the driver. Additionally, identifying methods for simulators to mimic physical stressors to that of authentic racing will provide an avenue for scientists and engineers to develop protocols to monitor driver fatigue in a more cost-effective manner compared to authentic racing.

Thus, the aim of the present investigation was to compare the physiological responses of drivers during authentic and simulated racing under similar environmental conditions (e.g., a hot cockpit). The hypothesis is that in the absences of elevated cornering G forces and psycho-emotional stress on the simulator there will be a blunted heart rate response and therefore, physiological strain index in the simulated racing group as compared to the authentic racing group.

## Methods

The experimental model of the current investigation compared physiological responses of authentic racing as compared to simulator racing. To appropriately model authentic racing, participants on the simulator wore the appropriate racing attire with the simulator located in an environmental chamber that artificially generated temperatures similar to the authentic race. The order of testing was randomized with the minimum time between testing being 2 weeks and the maximum time being 4 weeks. The time of day the authentic racing occurred was the same time of day used on the simulator settings. This study was approved by the Institutional Review Board at Michigan State University. All procedures performed were in accordance with the ethical standards established by the 1964 Declaration of Helsinki and its later amendments. All participants provided informed written consent prior to data collection.

### Participants

Authentic and simulated race data was collected from five licensed professional male racing drivers with at least 8 years of experience. The participants were 38.0 ± 5.1 (mean ± standard deviation) years old with an average height of 174.3 ± 3.9 cm, body mass of 81.2 ± 3.3 kg, and $\dot{V}O_{2\mathrm{peak}}$ of 54.1 ± 8.6 ml.kg^−1^.min^−1^ in accordance with the protocol outlined in McKnight, Bennett (3).

### Authentic racing data collection

Data was collected during the 2018 “Sahlen's 6 h at the Glen” race, located in Watkins Glen, NY. One lap of the racecourse is 5.47 km in length and includes 11 turns. The race started at 09:45 and lasted for 6 h. Five male participants were physiologically monitored while they drove Acura NSX GT3 race cars that competed in the IMSA GTD class.

The nature of GTD racing requires two drivers to alternate in shifts, with only one driver in the car at a time. Thus, drivers competed in 1 h stints over the course of the race. While each participant drove a total of 3 h only their first hour of driving was analyzed in order to be similar to the simulated racing and avoid cumulative effects of driving (15). The ambient temperature during the race was 33.8 ± 1.3°C with cockpit temperature being 41.3 ± 6.8°C measured every 10 min by a sensor fitted inside the cockpit at driver's helmet height to the centerline of the car. Participants wore required racing attire, which included a 3-layer Nomex® fire suit, gloves, race shoes, balaclava and helmet. Drivers consumed 10 ml·kg^−1^ per body weight of water 60 min before the start of the race to achieve euhydration. Following the start of the race, drivers consumed food and water *ad libitum*.

### Simulated racing data collection

**Figure 1:**
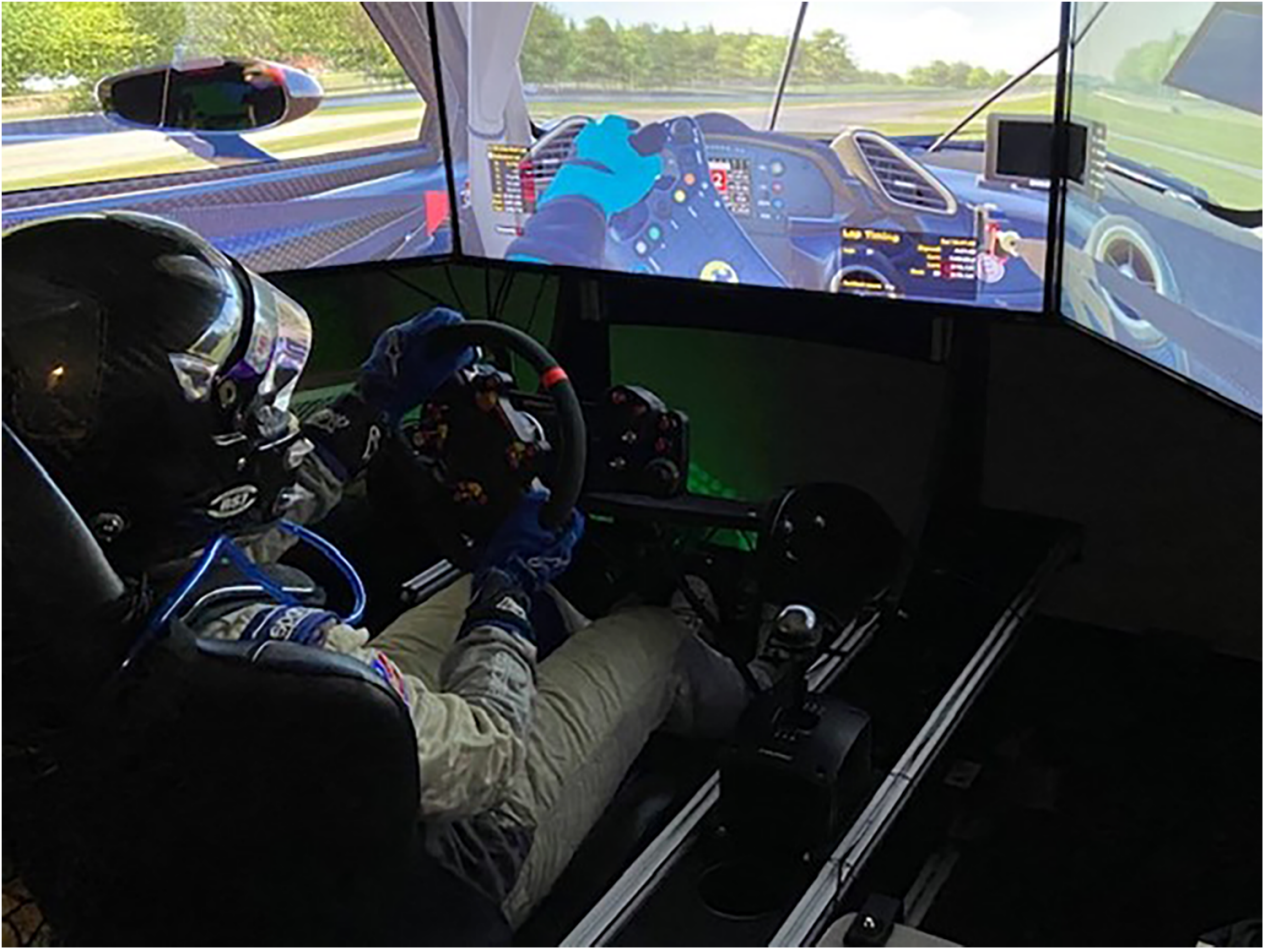
Configuration of the racing car simulator setup including screens, controls and racing attire.

The Spartan Motorsport Performance Laboratory has an AP-Xtreme Wide Triple Monitor racing simulator mounted on a gyroscope (Ricmotech, Miami Lakes, FL), which generates movements similar to those experienced in authentic racing (Figure 1). The simulator has a Ricmotech direct drive steering wheel and a Tilton GT3 Pro pedal assembly from an actual race car, including an accelerator and hydraulic brake pedal (Ricmotech). The combination of steering wheel and pedals elicits similar forces and feedback to those experienced during actual racing (16).

iRacing software (Bedford, Massachusetts), was run on the simulator with the participants driving on the Watkins Glen circuit in a Ferrari 488 GTD race car. iRacing at the time did not offer the Acura NSX GTD, but the Ferrari 488 elicits similar speeds and handling characteristics to the Acura, therefore was an appropriate model to replicate authentic racing. There were no other vehicles on the track during the simulation.

To ensure the simulator provided a realistic experience, a professional racing driver with over 20 years of experience, including victories at the 24 h of Daytona and the 24 h of Le Mans, set up the vehicle dynamics on the simulator. Consequently, the simulated race car responded to driver inputs as it would in actual racing, and importantly, the feedback (force on the steering wheel, pressure in the brake pedal, and rotation of the seat) from the simulator to the driver mimicked that of actual racing, except for the G forces placed on the driver's body during cornering, braking and vehicle vibration.

Fluid intake prior to commencement of the simulator was the same as that completed in the authentic racing. Participants drove the simulator for 60 min in a room heated to 38.0 ± 1.5℃, 40% relative humidity in the same racing attire as authentic racing. Participants were able to consume water *ad libitum*.

### Physiological measures

Physiological responses were recorded using a lightweight ambulatory monitoring system (Equivital EQ02, Hidalgo Ltd., UK). The Equivital LifeMonitor system recorded heart rate (HR; beats·min^−1^) breathing rate (breaths·min^−1^) and skin temperature (°C). Skin temperature was measured via an infrared sensor located within the Equivital LifeMonitor system on the right side of the body at the mid-axillary line. Core temperature (T_core_°C) measurements were obtained from an ingestible pill sensor (VitalSense, Mini Mitter, Philips Respironics, The Netherlands) at 15 s intervals. Ingestible pill sensors were swallowed at least three hours before the start of the first driving session in accordance with established guidelines to ensure good reliability and validity of measurement (17).

Participants wore the Equivital Life Monitor under their racing attire. All variables were recorded without interference from any race car electrical or communication systems.

Physiological Strain Index (PSI) was determined based on T_core_ (adjusted for core pill as opposed to rectal temperature) and HR using the following calculation (18):

$$\mathrm{PSI}=5(T_{\mathrm{coret}}-\;T_{\mathrm{core}0})\cdot (39.5-T_{\mathrm{core}0}{)}^{-1}+5(HR_{t}-HR_{0})\cdot (180-HR_{0}{)}^{-1}$$

In the equation, T_core*t*_ and HR*_t_* are simultaneous measurements taken during the data collection period, and T_core0_ and HR_0_ are the initial measurements taken once the racing attire was put on but before the race started (18).

### Statistics

An exploratory analysis was conducted to aid in the interpretation of the driver data to assist with a sensitivity analysis. A visual inspection of histograms and residual plots were used to determine normality of distribution. In the first 10 min of motor racing there is a rapid rise in physiological variables (15) due to increases in psychomotor stimulation and adjustment to environmental conditions. Therefore the 60 min of data collection was split into the first 10 min and the remaining 50 min.

A mixed linear model was used to determine interactions between time and racing trial (simulated or authentic). Fixed effects were assigned to trial and time (seconds) with participants assigned as random effects. Heart rate, skin temperature, core temperature and physiological strain index of authentic racing and simulated racing were analyzed by pairwise comparison with a Fishers LSD test adjustment for multiple comparison. Marginal *R*^2^ was used to estimate the variance explained by fixed factors (e.g., condition, time, condition x time). Analysis was completed in SPSS version 30 (IBM, Armonk, NY) with an alpha level set *a priori* of 0.05.

## Results

### Core body temperature

There was a main effect for trial, time and interaction (all *P* < 0.001; *R*^2^ = 0.041) for core temperature in the first 10 min. Pairwise comparisons revealed that core temperature was significantly (*P* < 0.001) greater in authentic racing compared simulated racing during the first 10 min. The clinical significance of this difference is negligible given the mean ± standard deviation temperature of authentic and simulated racing was 37.6 ± 0.02°C and 37.5 ± 0.03°C respectively (Table 1).

**Table 1:** Physiological parameters whilst undertaking authentic racing (AR) and simulated racing (SR).

Parameter	Condition	First 10 min	Final 50 min
Core temperature (°C)	AR	37.6 ± 0.3*	38.5 ± 0.6*
	SR	37.5 ± 0.4	37.4 ± 0.3
Heart Rate (beats·min^−1^)	AR	125 ± 23*	159 ± 23*
	SR	112 ± 15	112 ± 19
Percentage Heart Rate Max (%)	AR	67.1 ± 12.5	85.3 ± 12.5*
	SR	60.0 ± 7.6	59.9 ± 8.3
PSI (A.U)	AR	3.1 ± 1.4	7.3 ± 2.5*
	SR	1.1 ± 0.9	0.7 ± 1.1
Skin Temperature (°C)	AR	36.7 ± 0.5*	37.6 ± 1.5
	SR	35.7 ± 1.0	37.0 ± 0.5
Breathing Rate (breaths·min^−1^)	AR	28.8 ± 6.8	34.8 ± 6.5*
	SR	24.0 ± 4.3	21.7 ± 3.8

* Difference between trials (*P* < 0.05). Values are reported as mean ± SD.

**Figure 2:**
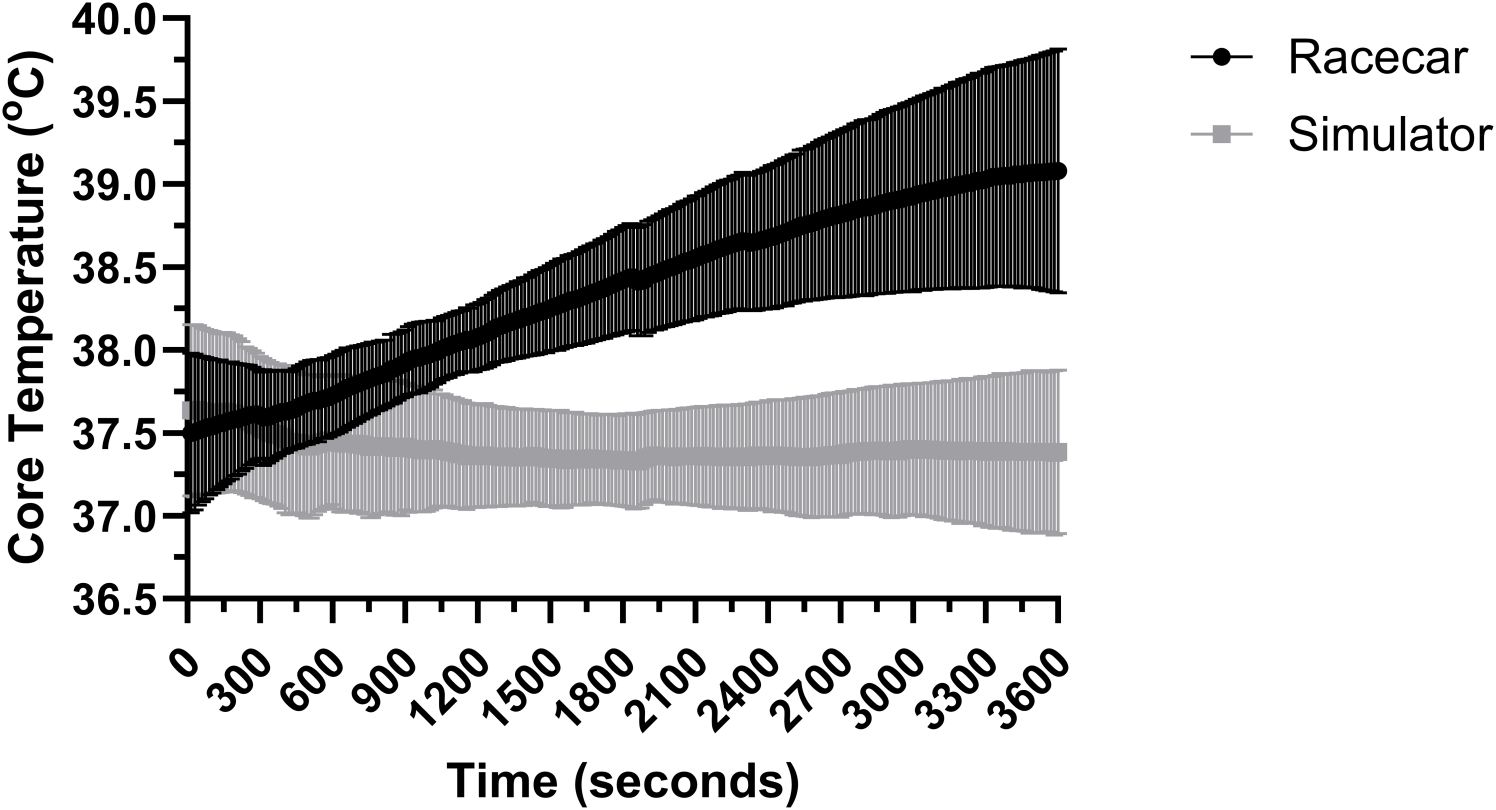
Core temperature over the full 60 min of simulated and authentic racing, including the initial 10 min and the remaining 50 min combined (mean ± SD).

For core temperature in 50 min following the initial exposure there was an effect for trial, and overall interaction effect (both *P* < 0.001; *R*^2^ = 0.744) however, there was no effect for time (*P* = 0.920). Pairwise comparisons indicated that authentic racing was 1.14 ± 0.01°C (mean ± standard error) higher than simulated racing during the final 50 min (*P* < 0.001). Core body temperature data across the 60 min of racing is presented in Figure 2.

### Heart rate

**Figure 3:**
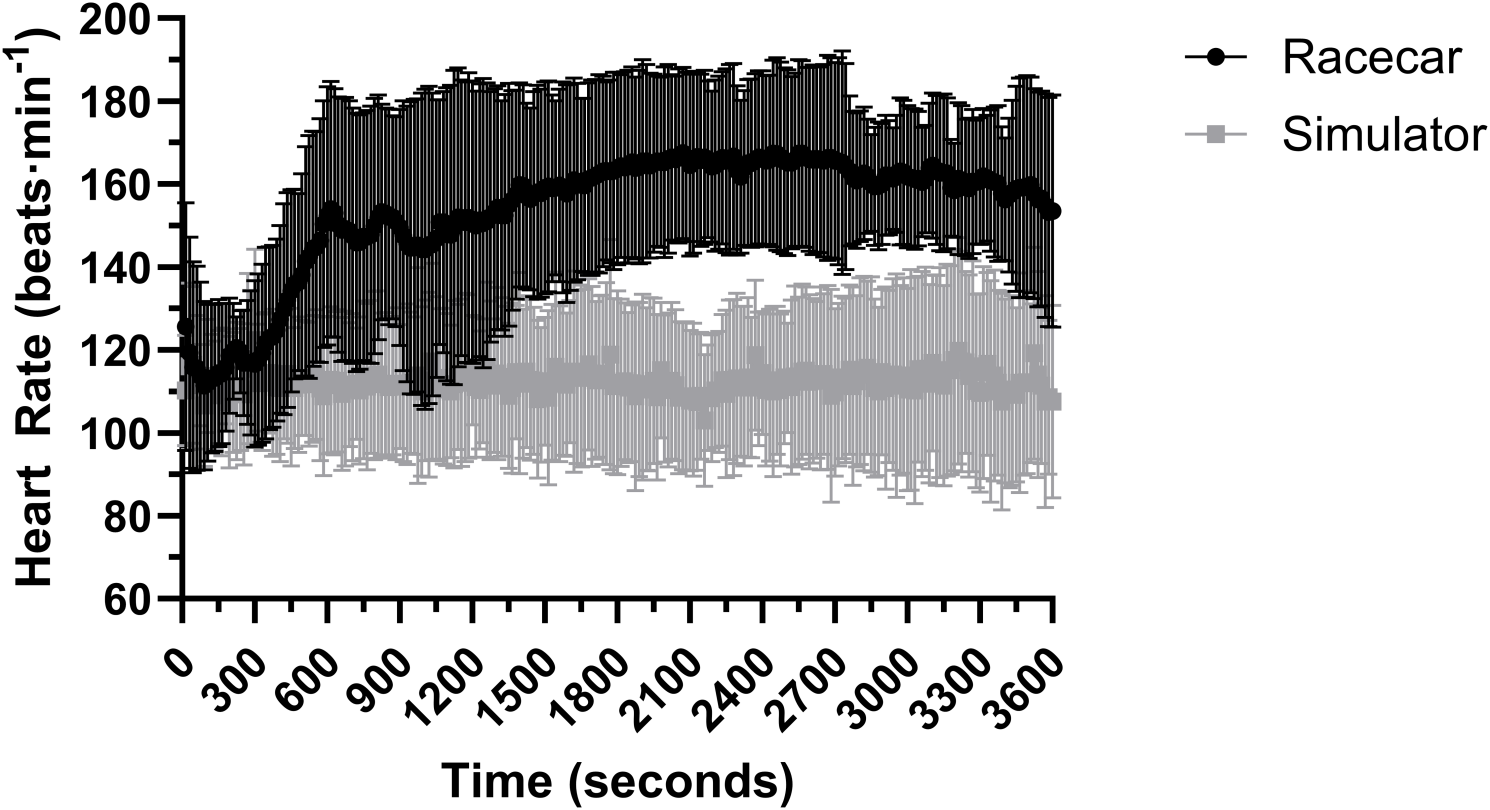
Heart rate over the full 60 min of simulated and authentic racing, including the initial 10 min and the remaining 50 min combined (mean ± SD).

Between authentic and simulated racing there was no effect for time on heart rate in 10 min (*P* = 0.832) and 50 min (*P* = 0.536) of racing. There was an effect for trial and interaction in both 10 min (both *P* < 0.05; *R*^2^ = 0.203) and 50 min of racing (both *P* < 0.001; *R*^2^ = 0.530). Pairwise comparisons indicate that heart rate was 13 ± 1 beats·min^−1^ higher in authentic racing compared to simulated racing (*P* < 0.001) for the first 10 min. Heart rate in the first 10 min of racing is presented in Figure 3. Heart rate continued to rise during the final 50 min of authentic racing, exceeding that of simulated racing by 47 ± 1 beats·min^−1^ (*P* < 0.001). In contrast, heart rate in the simulator showed no significant rise compared to the levels recorded in the first 10 min of racing. Heart rate for the entire race duration is presented in Figure 3. Mean, standard deviations are presented in Table 1 for heart rate and percentage heart max.

### Physiological strain index

**Figure 4:**
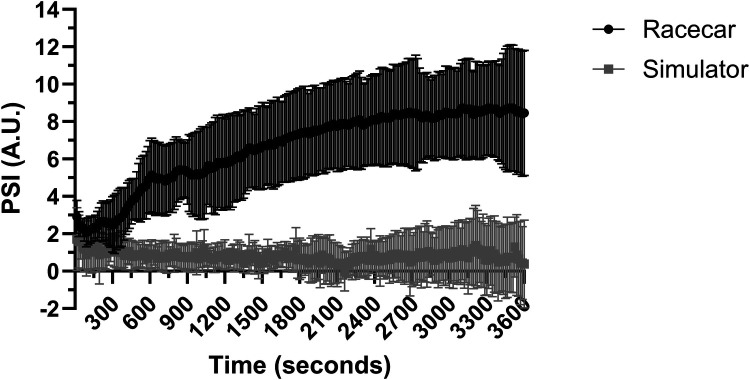
Physiological strain index (PSI) over the full 60 min of simulated and authentic racing, including the initial 10 min and the remaining 50 min combined (mean ± SD).

There was a significant effect for time (*P* = 0.013) and interaction (*P* < 0.001; *R*^2^ = 0.536), however there was no significant effect for trial (*P* = 0.104) in the first 10 min of racing (Figure 4). In the final 50 min there was a main effect for trial and an interaction effect (both *P* < 0.001; *R*^2^ = 0.768) however, there was no effect for time (*P* = 0.757). Pairwise comparisons revealed that authentic racing was greater than simulated racing throughout the final 50 min of racing (*P* < 0.001). In the simulated racing PSI remained relatively unchanged in the final 50 min from the initial 10 min of racing (Figures 3, 4).

### Skin temperature

**Figure 5:**
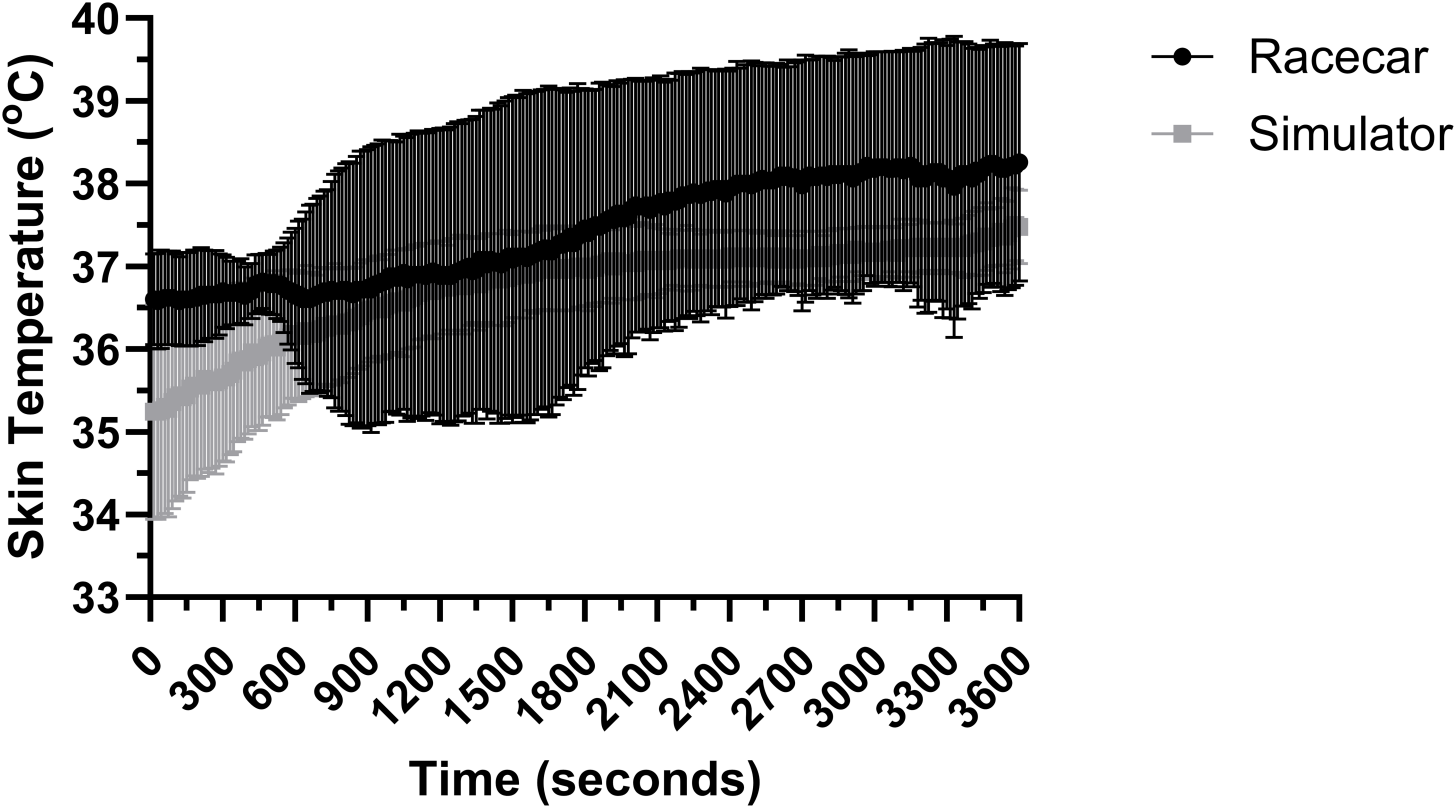
Skin temperature over the full 60 min of simulated and authentic racing, including the initial 10 min and the remaining 50 min combined (mean ± SD).

A significant (all *P* < 0.001; *R*^2^ = 0.318) effect for time, trial and interaction effect was found between authentic racing and simulated racing conditions on skin temperature in the first 10 min. Skin temperature across 10 min of authentic and simulated racing is presented in Figure 5. In the final 50 min of racing there was an effect for time and interaction effect (both *P* < 0.001; *R*^2^ = 0.181), however there was no effect for trial (*P* = 0.928). Skin temperature across the final 50 min of authentic and simulated racing is presented in Figures 4, 5.

### Breathing rate

There was no significant effect for trial (*P* = 0.695) or time (*P* = 0.465) on breathing rate during the first 10 min of racing. However, there was a significant interaction effect (*P* < 0.001; *R*^2^ = 0.211) between trial and time for breathing rate in the first 10 min of racing. Conversely, there was no trial and time interaction effect in the final 50 min of racing (*P* = 0.158; *R*^2^ = 0.596). Trial and time effects alone were significant (both *P* < 0.05) in the final 50 min of racing. Pairwise comparisons showed a significantly (*P* < 0.001) higher breathing rate in authentic compared to simulated racing.

## Discussion

The purpose of this study was to observe a drivers' physiological response between simulated and authentic racing. The results showed that in the absence of G-loading, psycho-emotional stress and competitive pressures, heart rate, core temperature, breathing rate and physiological strain index are not replicable in a simulated environment. After a short period (10 min) of racing there is a considerable increase in metabolic work, psycho-emotional stress, competitive pressure and physical stress required in authentic racing compared to simulated racing.

Authentic racing and simulated motor-racing elicited no significant difference in skin temperatures, likely due to the heat protocol for the simulator mirroring that of authentic motor-racing, with drivers in both environments wearing a standard racing suit and fireproofs underneath.

However, drivers in the authentic race car had a higher core body temperature than those driving the simulator. In the simulator, there is a reduction in metabolic work needed to drive the race car, and it is known that increased metabolic work increases core temperature (19). The elevated metabolic work of race car drivers is a result of increased skeletal muscle contraction to (1) pilot the vehicle (steering and pressing the pedals) and (2) resisting the gravitational forces. As the simulator required identical force to pilot the vehicle as authentic racing, the increase in metabolic work for drivers in the authentic race car is a result of skeletal muscle contraction needed to resist the gravitational forces that occur during cornering maneuvers (10). As such, the reduced metabolic work in the simulator does not increase core temperature and does not stress the body in the same way that authentic racing does.

Two important findings from the study are that heart rate and PSI are increased in authentic motor-racing as compared to simulated racing. Heart rate and PSI have been regularly evaluated in existing motorsport literature with increases in heart rate (up to 85% heart rate maximum) observed during motor-racing (2, 6–10, 15). There are two possible scenarios for reduced heart rate and PSI in simulated racing are (1) the simulator is less physically demanding (as described above) and (2) the simulator elicits a reduced psycho-emotional and competitive state. In 1987, Schwaberger was the first to observe that racing drivers had an increased catecholamine response when sitting in the race car and concluded that psycho-emotional stress contributted to the elevations in heart rate (and indirectly PSI) during authentic racing (11). The present investigation supports the initial findings with observations that heart rate increased once the driver sat in the race car and increased over the first 10 min (Figure 3). Elevations in heart rate have been reported to occur in the authentic racing when there was minimal physical work or thermal straining, indicating a psycho-emotional component (10, 11, 15). Drivers racing in authentic scenarios experience higher psycho-emotional stress and competitive pressure due to a variety of factors such as: injury risk, hindering weather conditions, distraction from fans, and competitive pressures (11). These variables are not present in simulated settings. Other simulated racing studies have shown similar heart rate responses (peak: 126 ± 19 bpm) in hot environments (50°C) during 60 min of racing however, our study had lower PSI and core temperature changes most likely due to the different temperatures used in the studies (20). The heart rate elevations in the first 10 min did not occur in the simulated scenario indicating the absence/reduction of a psycho-psycho-emotional response and reduced physical load.

Automobile racing is a physically demanding sport that requires drivers to compete at 65%–85% of their maximal aerobic capacity (21). G-forces alone have been attributed to increase in heart rate to 120–140 beats·min^−1^ under +4 Gz which is not uncommon in some motorsport events where G-force may be >5.5 Gz (22, 23). Highest g-forces are observed during acceleration, deceleration (braking) and cornering with the subsequent physical effect of increased weight and limited range of motion. This contributes to greater mechanical work and therefore metabolic work to pilot the vehicle safely and as fast as possible. The physical demands from g-force exposure in authentic racing may in part explain higher core temperatures, heart rates and breathing rates through higher mechanical demands.

A unique aspect of automobile racing is that the driver and car performance must be optimized to achieve success during competition. With the advent of computer technology racing teams are now able to develop computerized driving simulators. Simulated motor-racing has become an increasingly popular training platform for motorsport athletes, as it allows for repeated testing in a safe environment which allows for optimal car setup and understanding of the “driving line for optimal track speed”. In fact, for a 75 lap Formula 1 Grand Prix race most teams will have their drivers complete 500 laps on the simulator before arriving at the race venue (1). As the cost of racing has increased and there are limited testing days available, teams have begun to rely more on simulators over authentic practice sessions. This development has allowed engineers to optimize the car setup and drivers are able to learn the track and perfect their race craft before arriving at the actual race venue for competition (24). Simulators provide many benefits to drivers, such as a safe environment that eliminates the risks associated with an actual race car (6). However, a racing simulator does not generate the physical loads to drive the car or the psycho-emotional stress and competitive pressures of the racing event. Simulators have begun to utilize direct drive steering wheels and hydraulic pedals that require users to elicit more force in an effort to simulate the g-forces of an actual race car. Although simulators provide safe and controlled environments, the absolute validity of varying simulators (high fidelity and low fidelity) is yet to be undertaken in a motor-racing setting (25) Race car simulators offer a unique opportunity for training drivers in critical behaviours that enhance performance, such as visual scanning of the racetrack and strategic car positioning. The ecological framework highlights the interplay between perception and action, emphasizing that drivers must not only perceive to move but also move to perceive effectively (26–28). For instance, the way drivers position their cars on the track can influence their field of view and their ability to detect actionable affordances (27). Simulators provide a controlled environment to study these dynamics by recording car position and gaze behaviour under various racing and overtaking scenarios. While promising, further research is needed to explore the transferability of such simulator-based training to real-world racing contexts.

## Limitations

As with most studies, the design of the current investigation is subject to limitations that could be addressed in future research. The first limitation is that, despite our best efforts, we were unable to control all confounding variables in this investigation. The driver's dietary intake and fluid intake couldn't be controlled in the authentic racing. It is important to note that fluid consumption was controlled during simulated racing data collection, however, in the authentic racing drivers consumed a fluid volume to ensure euhydration when the core temperature pills were ingested but for the remainder of data collection drivers consumed fluid *ad libitum*. The second limitation is the small sample size of five participants. In field-based motor racing studies sample sizes of three (15, 29) to eight (30) participants are commonly reported. Our sample is in alignment with previous research and is the first study that has used replicated laboratory and field data collection methods in motor racing. Another limitation of this study is that skin temperature was only measured at a single site, as the only system approved for physiological monitoring by the Fédération Internationale de l'Automobile (FIA) is the Equivatal system, which uses infrared sensors and does not align with the standards set forth in ISO 9886:2004 of multi-site measurement for mean-weighted skin temperature. Finally, the simulator temperature was lower than in authentic racing to be compliant with FIA regulation 901-1(8), which may have resulted in lower physiological responses compared to real-world conditions.

## Conclusion

Overall, the findings from our study indicate that driving a race car is more physically demanding than driving a simulator, as evidenced by the higher heart rate, core temperature, and physiological strain index observed in race car drivers. Differences in physiological demands become more apparent after 10 min of racing in a 60 min race. In certain circumstances simulated motor-racing environments should aim to mimic those experienced by drivers to represent authentic motor-racing.

## Data Availability

The raw data supporting the conclusions of this article will be made available by the authors, without undue reservation.
